# Supplementary material for: High-resolution spatiotemporal transcriptome mapping of tomato fruit development and ripening
Source: Nat Commun. 2018 Jan 25;9:364. doi: 10.1038/s41467-017-02782-9 (PMC5785480; doi:10.1038/s41467-017-02782-9)
Supplement: Supplementary file 1 — Supplementary Information [file 41467_2017_2782_MOESM1_ESM.pdf]

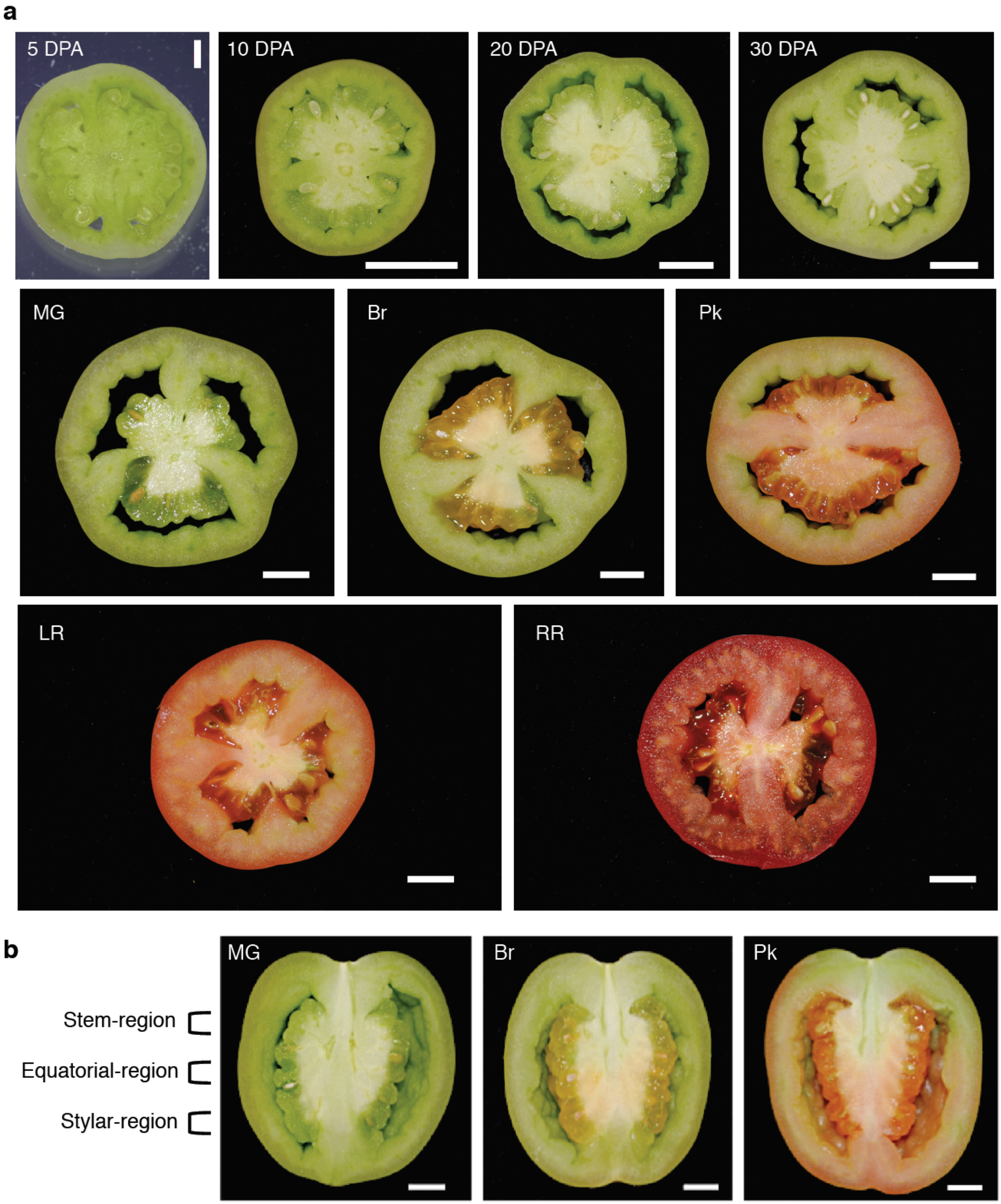

Supplementary Fig. 1 Representative pictures of fruit sections. **a** Equatorial sections of fruit at the developmental stages used for tissue harvest. **b** Vertical sections at early ripening stages indicating three latitudinal regions used for tissue harvest. Scale bars: 1 mm for 5 DPA, 1 cm for 10 DPA–RR. DPA, days post anthesis; MG, mature green; Br, breaker; Pk, pink; LR, light red; RR, red ripe.

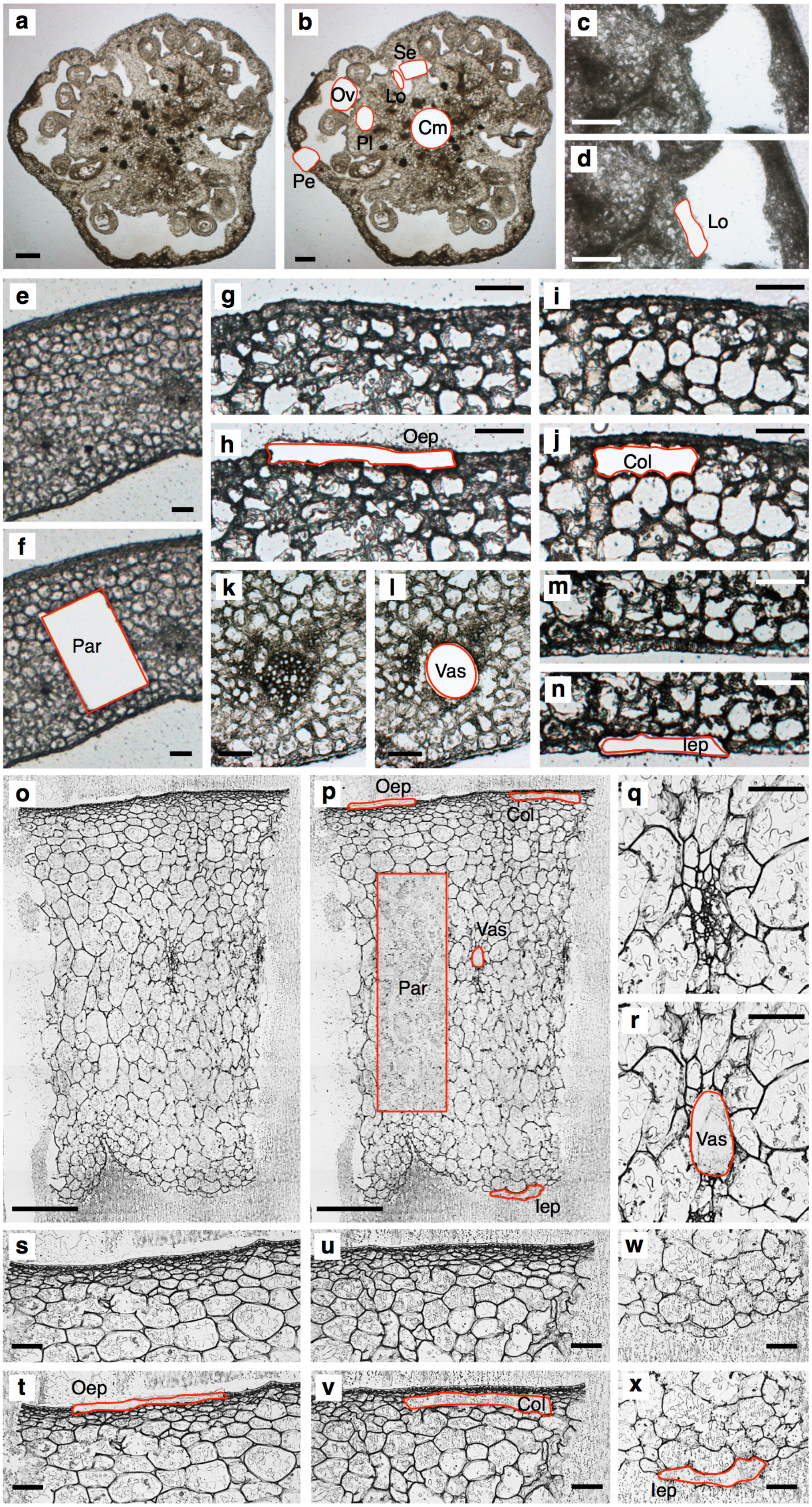

Supplementary Fig. 2 Representative section images of tissues and cells collected by LM. **a**–**x** Examples of sections of ovaries at anthesis **a**–**d** and pericarp at 5 days post anthesis **e**–**n** on membrane slides and MG pericarp **p**–**x** on glass slides. **a**, **c**, **e**, **g**, **i**, **k**, **m**, **o**, **q**, **s**, **u** and **w** show sections before LM, and **b**, **d**, **f**, **h**, **j**, **l**, **n**, **p**, **r**, **t**, **v** and **x** show sections after LM. Scale bars: in 100 µm in **a**–**d** and **q**–**x**, 50 µm in **e**–**n**, 1,000 µm in **o** and **p**. Cm, columella; Lo, locular tissue; Ov, ovule; Pe, pericarp; Pl, placenta; Se, septum; Col, collenchyma; Iep, inner epidermis; Oep, outer epidermis; Par, parenchyma; Vas, vascular tissue.

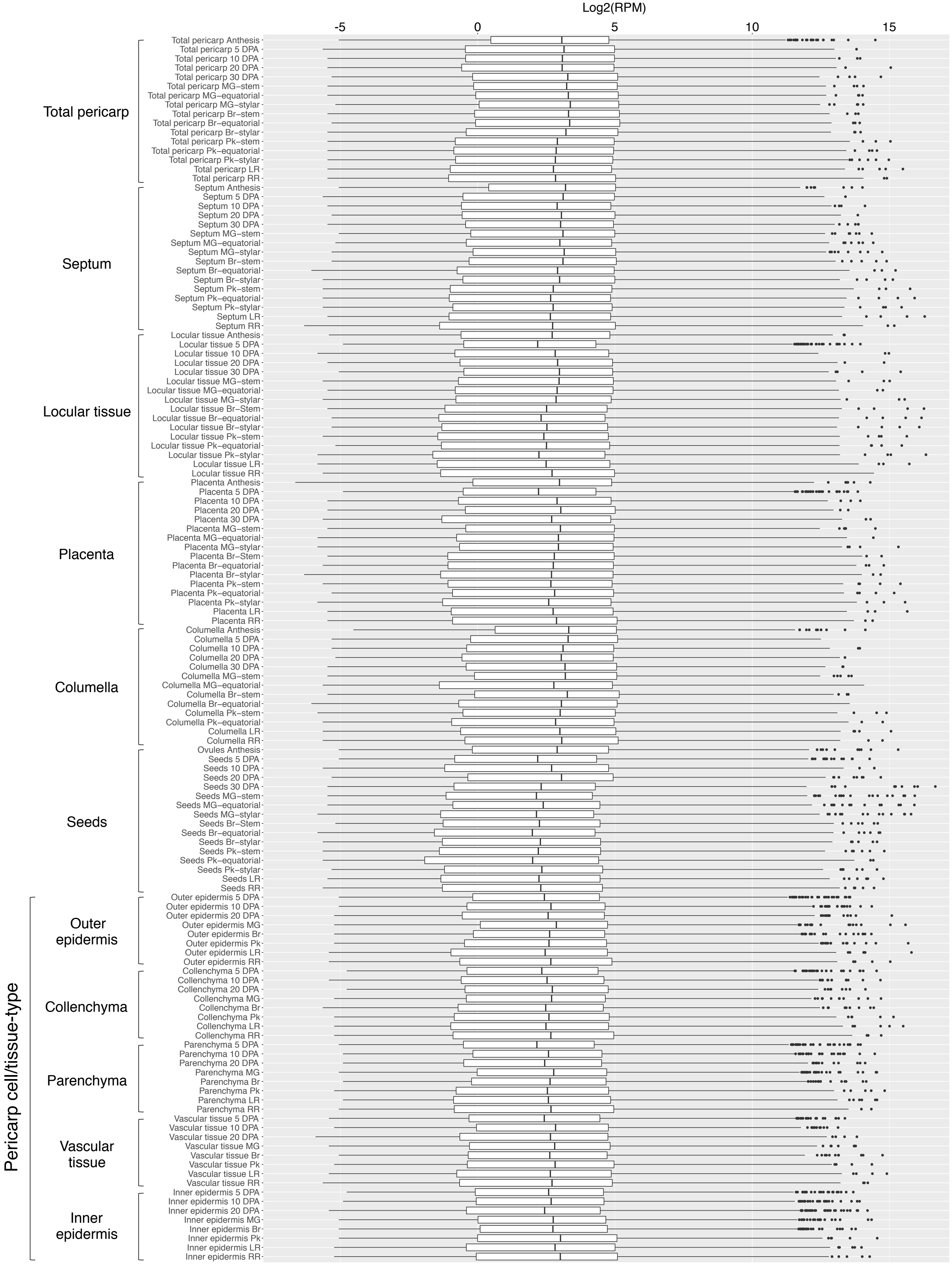

Supplementary Fig. 3 Distribution of RPM normalized expression. Boxplots show the interquartile range (IQR) of averaged RPM values in log2-scale with the median (a vertical line within IQR), data range (horizontal line) and outliers (dots).

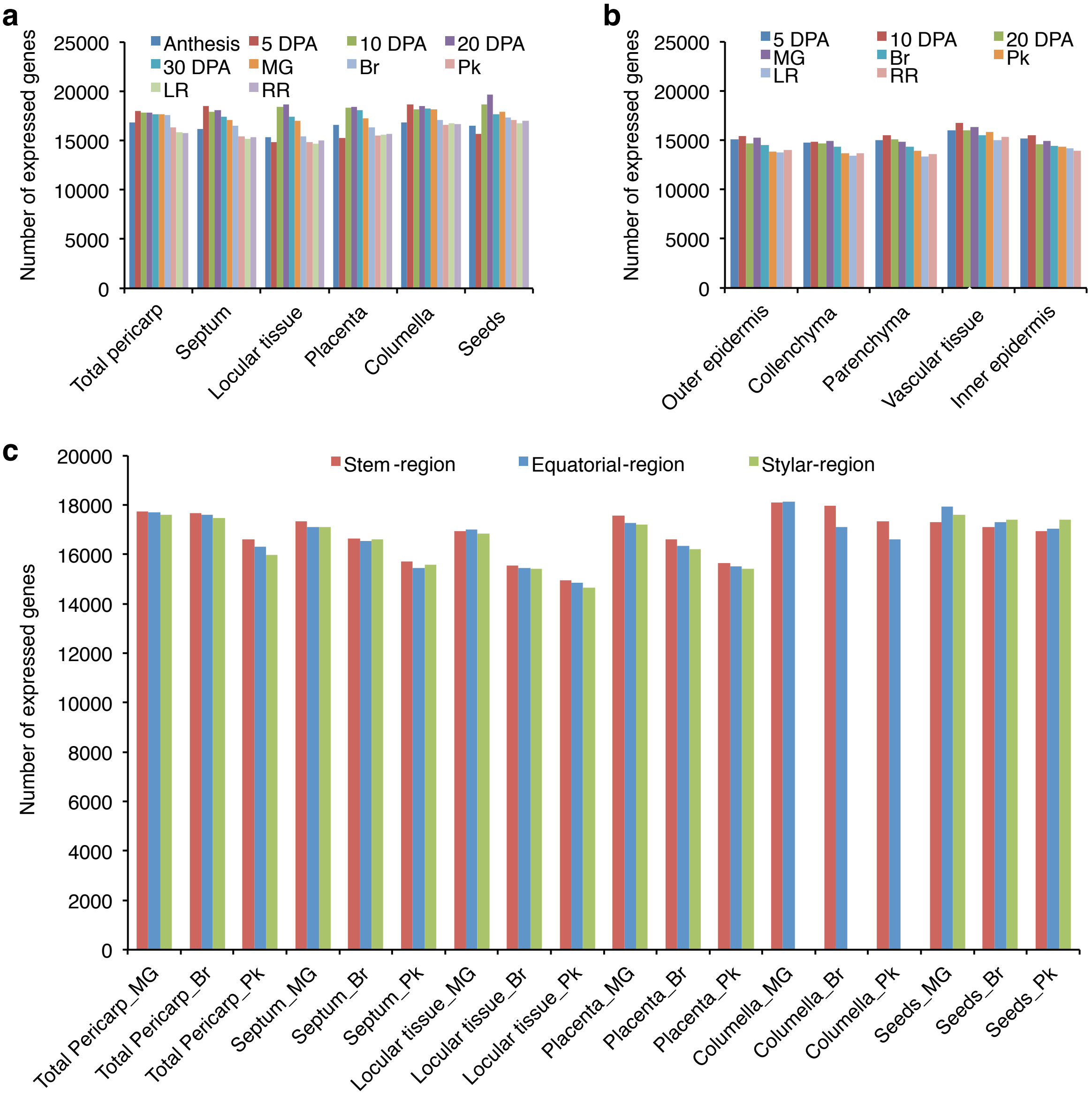

Supplementary Fig. 4 Number of expressed genes in fruit tissue/cell-types. **a**, **b** Bars show number of expressed genes at individual developmental stages in fruit major tissues from equatorial regions **a**, pericarp cells **b** and major tissues from three different latitudinal sections of fruit **c**. Columella tissues were harvested only from the equatorial-region and stem-region. DPA, days post anthesis; MG, mature green; Br, breaker; Pk, pink; LR, light red; RR, red ripe.

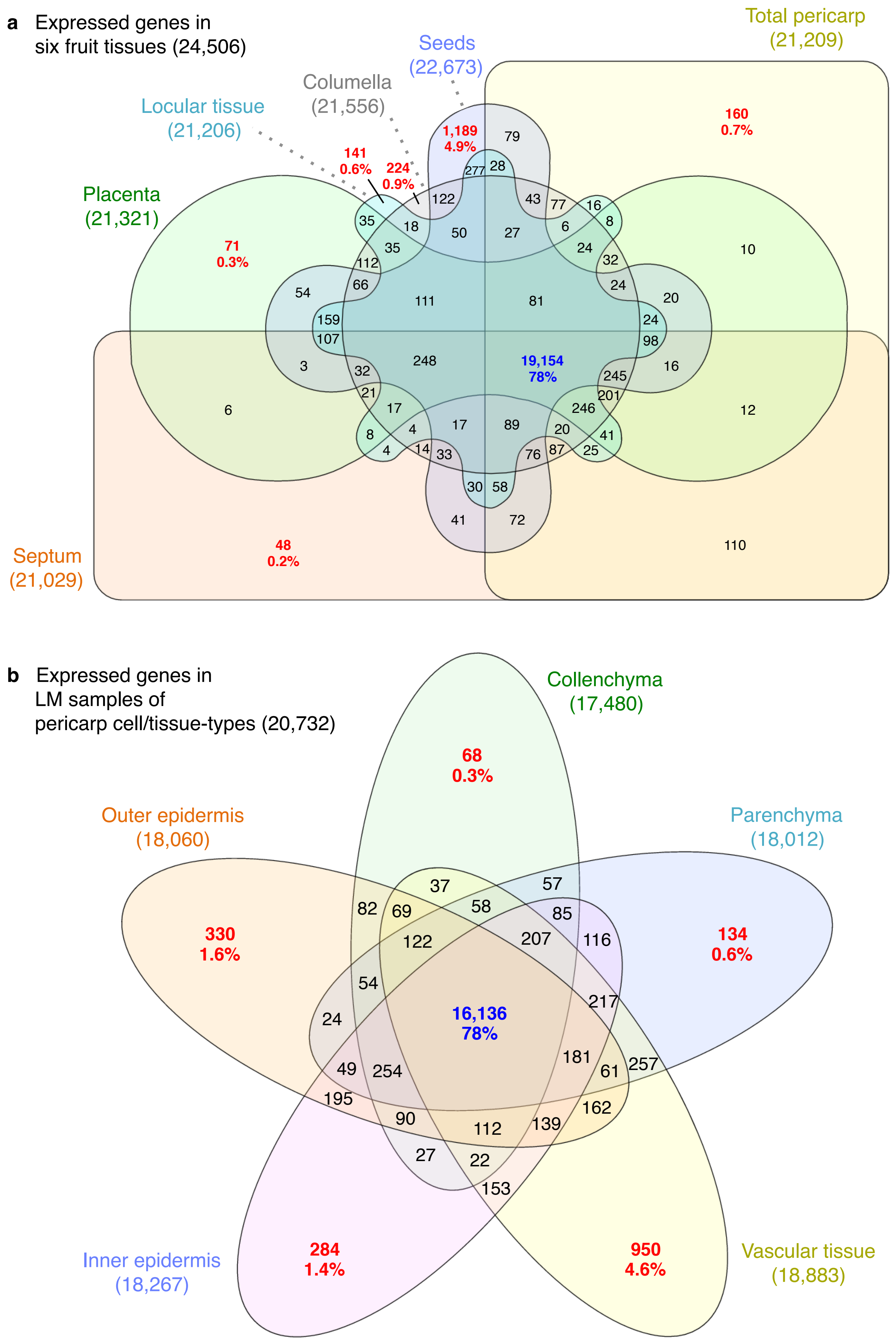

Supplementary Fig. 5 Distribution of expressed genes among fruit tissue/cell-types. **a, b** Venn diagram of the numbers of genes expressed in five fruit component tissues **a** and pericarp cells/tissues **b** from the equatorial-region across the tested developmental stages. Numbers with percentages in blue and red indicate genes expressed in all major fruit tissues or pericarp cells, and in a specific tissues or cells, respectively. The numbers of genes included are shown in parentheses.

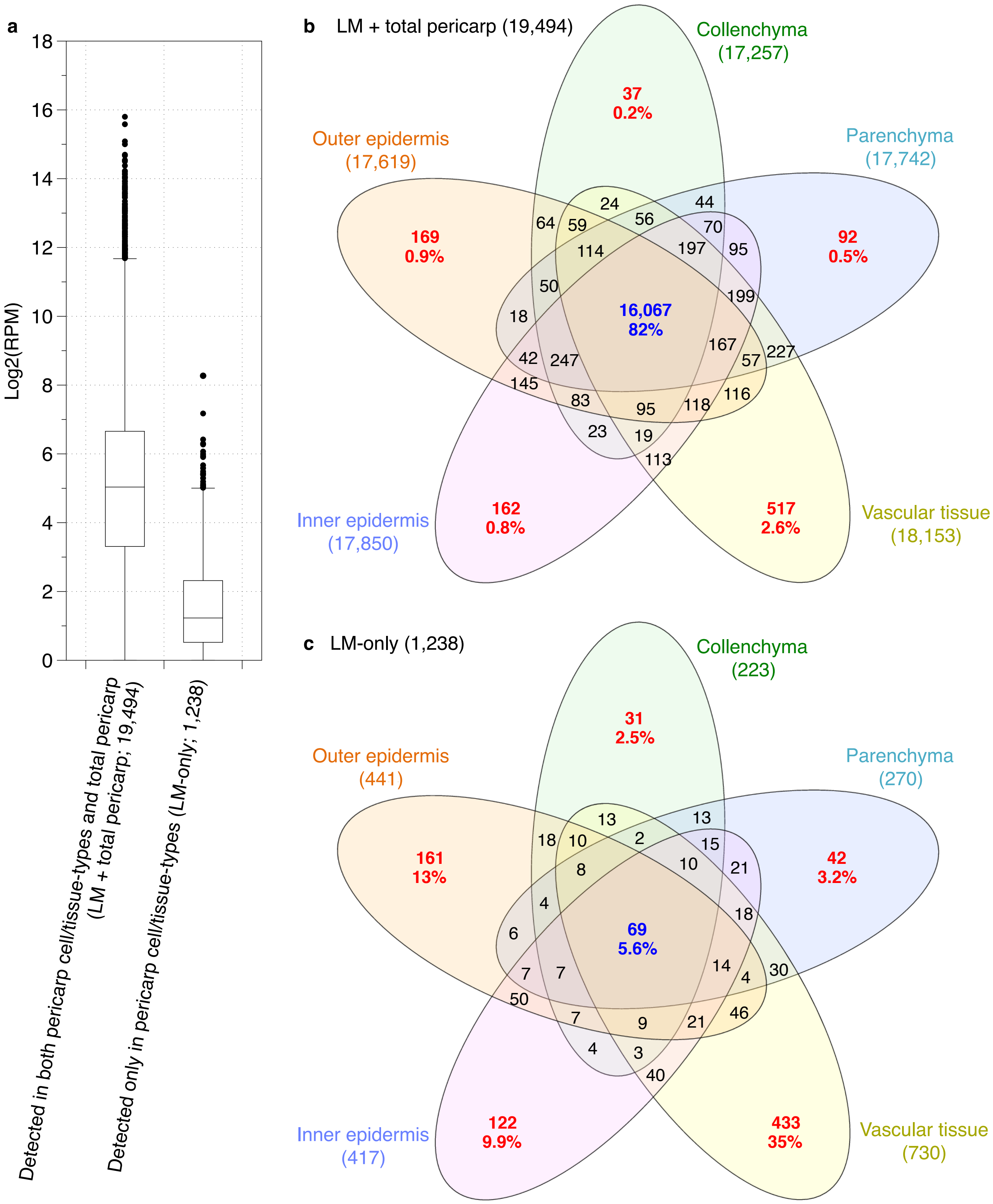

Supplemental Fig. 6 Expression profile of genes identified through LM. **a** Distribution of maximum RPM values of genes detected in both pericarp cell/tissue-types isolated by LM and total pericarp (LM + total pericarp), and genes detected in cell/tissue-types via LM, but not in total pericarp (LM-only). Boxplots show the interquartile range (IQR) of the maximum of averaged values throughout development in log2-scale with the median (a horizontal line within IQR), data range (vertical line) and outliers (dots). **b**, **c** Venn diagram of the numbers of genes categorized as LM + total pericarp **b** and LM-only **c**. Numbers with percentages indicated in blue and red correspond to genes expressed in all pericarp cell/ tissue-types and in a specific cell/tissue-type, respectively. The numbers of genes included are shown in parentheses.

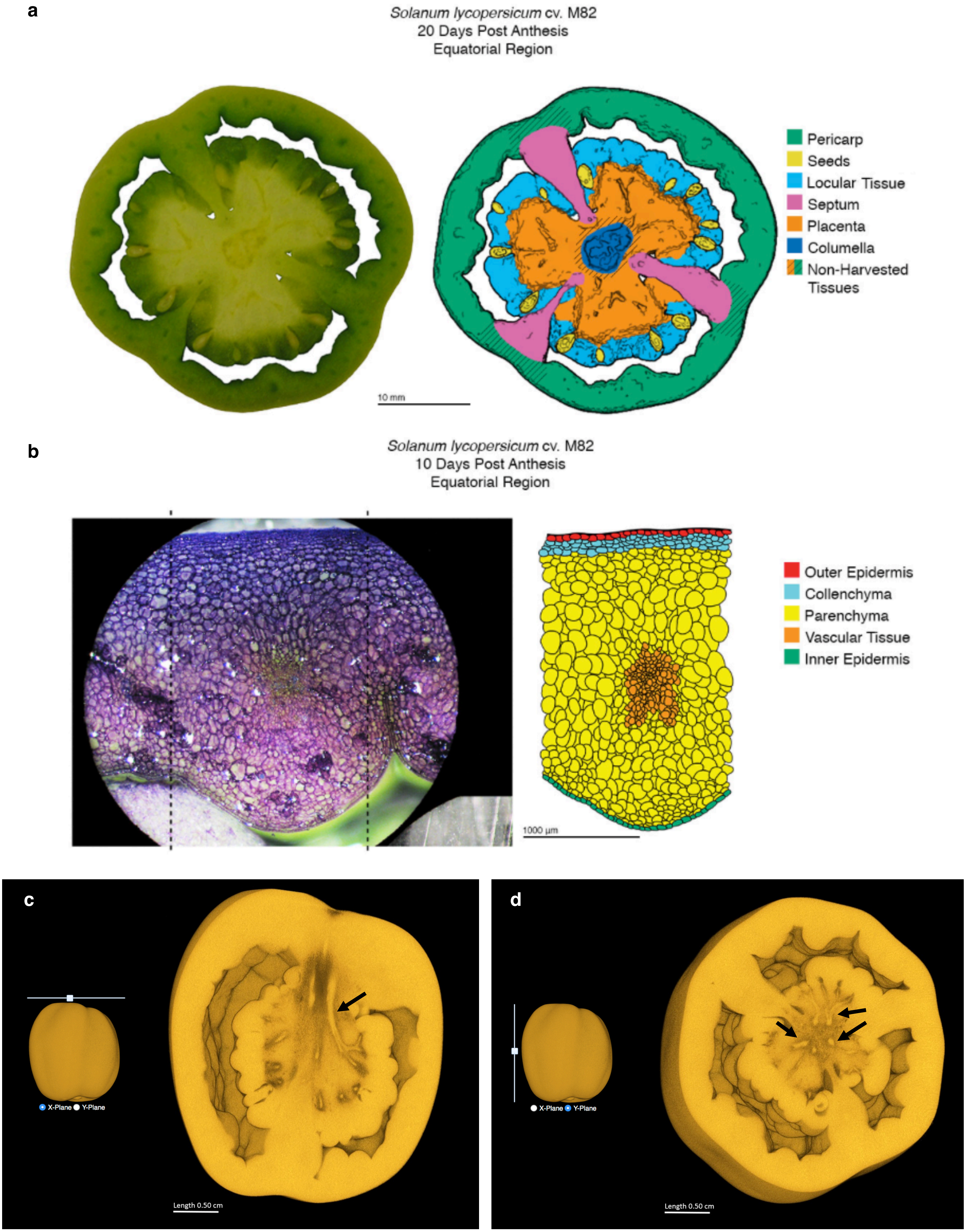

Supplementary Fig. 7 Anatomical structures of tomato fruit tissues shown in the Anatomy Viewer function of the Tomato Expression Atlas database. **a**, **b** A fruit section (left) and a traced and colorized light microscope image (right) are shown, highlighting differnt tissues and cells of the fruit **a** and pericarp **b** targeted from gene expression analyses. **c**, **d** Three-dimentional reconstruction of computed tomography images of mature green fruit, showing internal structures. The interface allows users to observe internal structures of representative 10 days post anthesis (DPA), 15 DPA, 20 DPA, 30 DPA, mature green, breaker and pink fruit from vertical **c** and horizontal plane **d** with different depth, as well as video clips of the fruit. Arrows indicate representative vascular tissues in collenchyma and placenta.

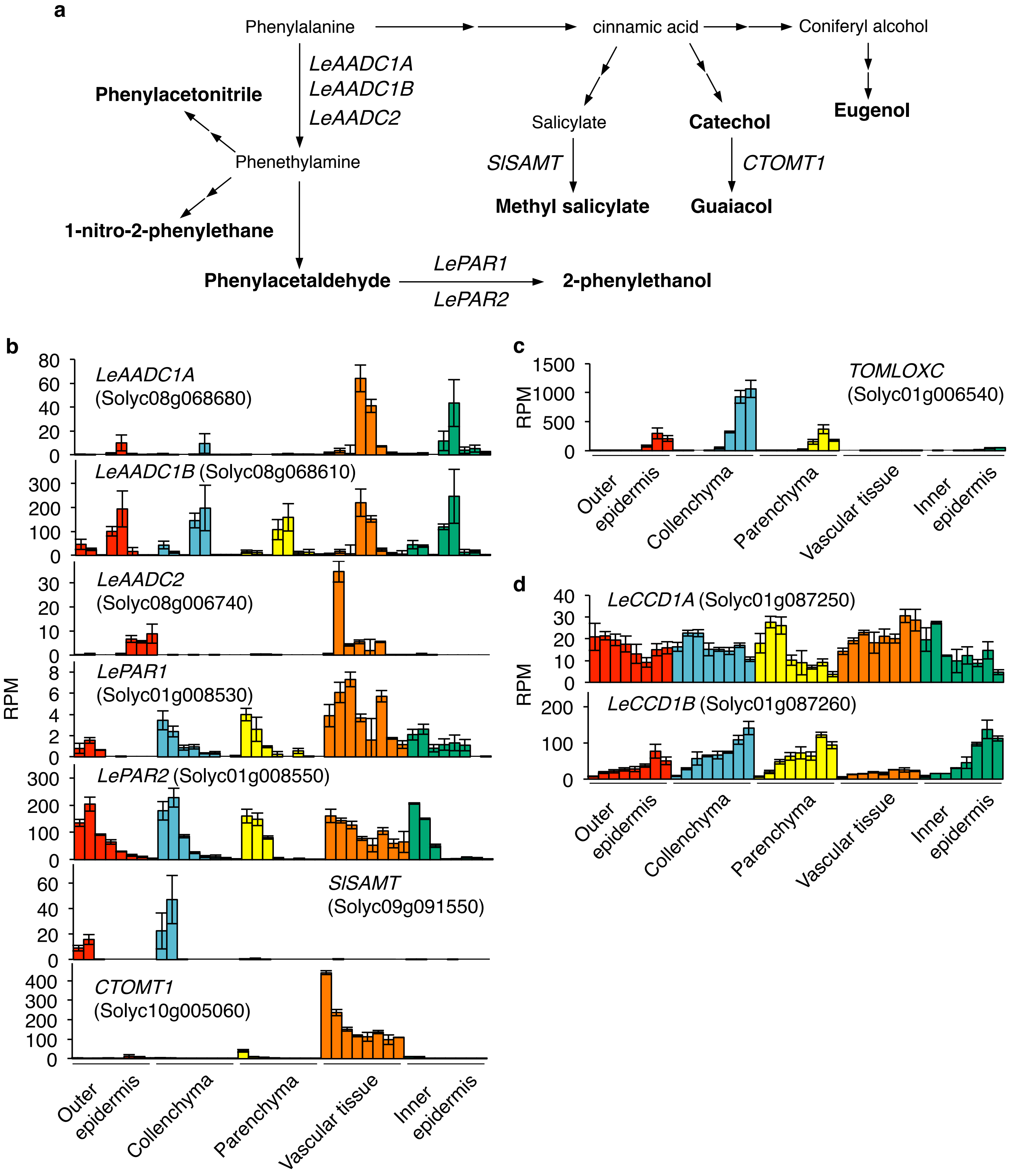

Supplementary Fig. 8 Pericarp cell/tissue-based expression of genes associated with the biosynthesis of volatile compounds. **a** Overview of the phenolic volatile biosynthetic pathway in tomato fruit. Major volatile compounds in tomato fruit are represented in bold. **b–d** Characterized genes involved in the pathway are italicized. RPM expression values of selected genes involved in biosynthesis of volatiles derived from phenylalanine **b**, fatty acids **c**, and carotenoids **d**. Colored bars indicate mean RPM ± s.e.m. (n = 3) in each cell/tissue-type at each stage (left to right; 5 days post anthesis (DPA), 10 DPA, 20 DPA, mature green, breaker, pink, light red and red ripe).

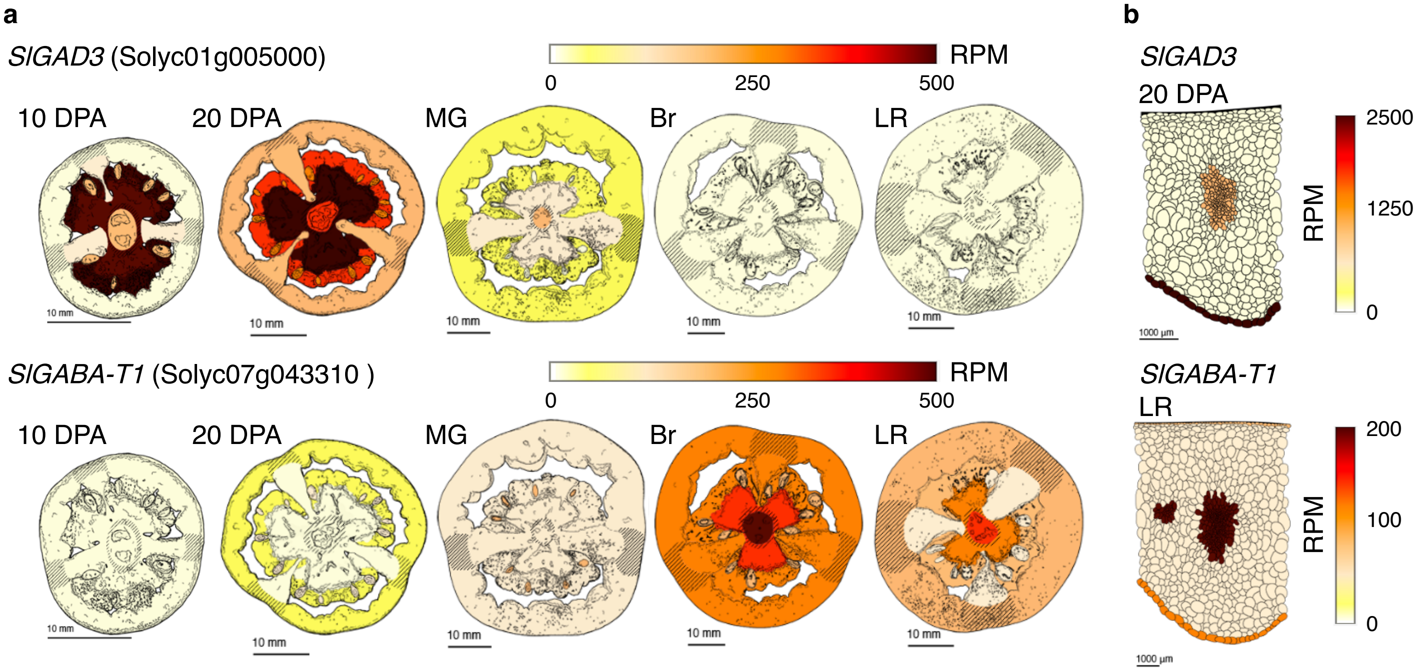

Supplementary Fig. 9 Spatiotemporal expression patterns of genes associated with GABA accumulation. **a**, **b** Fruit tissue- **a** and pericap cell/tissue-based **b** expression images at selected developmental stages generated through the Tomato Expression Atlas (TEA) database are shown.

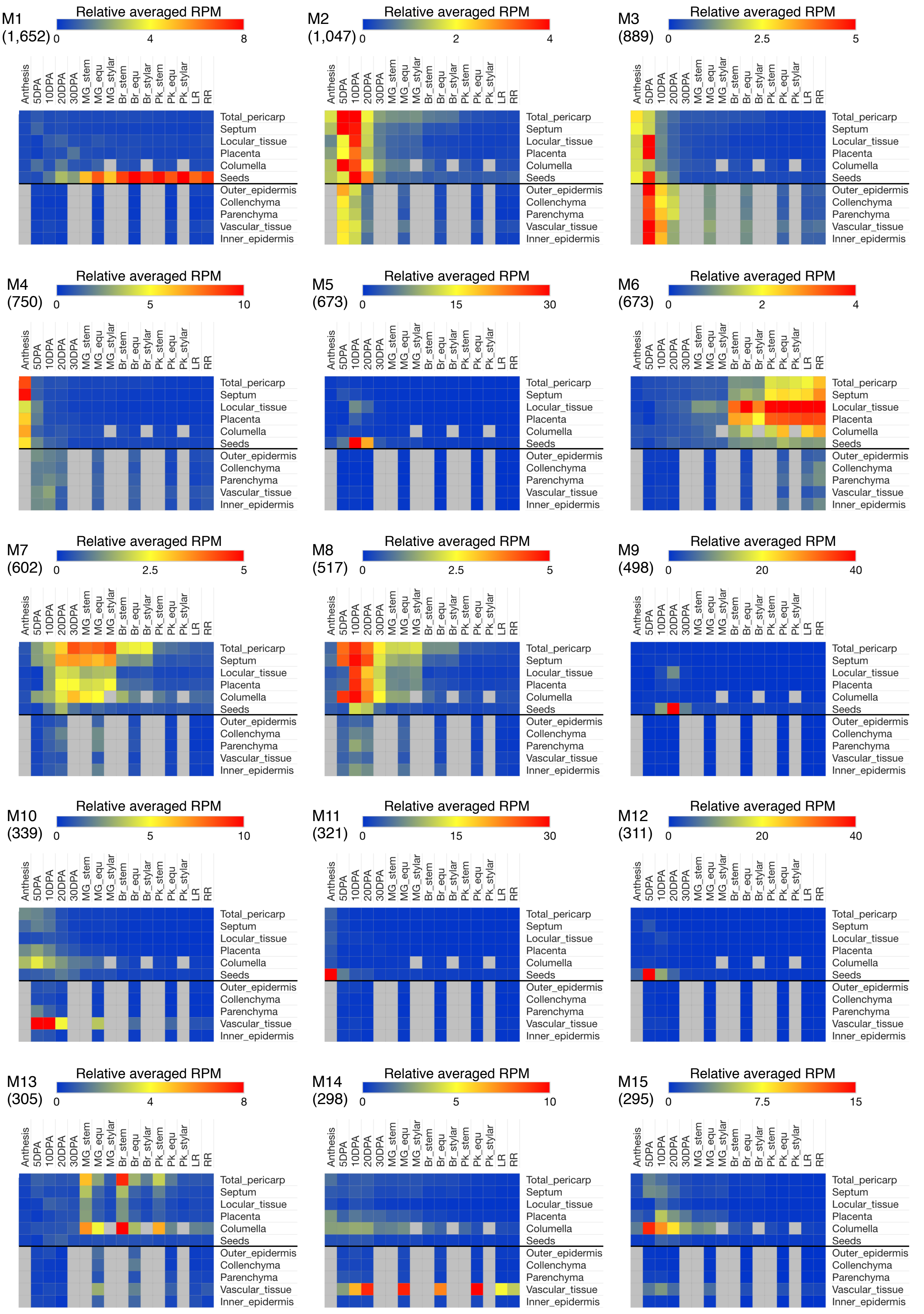
M1
(1,652)
Relative averaged RPM
0
4
8
M2
(1,047)
Relative averaged RPM
0
2
4
M3
(889)
Relative averaged RPM
0
2.5
5
M4
(750)
Relative averaged RPM
0
5
10
M5
(673)
Relative averaged RPM
0
15
30
M6
(673)
Relative averaged RPM
0
2
4
M7
(602)
Relative averaged RPM
0
2.5
5
M8
(517)
Relative averaged RPM
0
2.5
5
M9
(498)
Relative averaged RPM
0
20
40
M10
(339)
Relative averaged RPM
0
5
10
M11
(321)
Relative averaged RPM
0
15
30
M12
(311)
Relative averaged RPM
0
20
40
M13
(305)
Relative averaged RPM
0
4
8
M14
(298)
Relative averaged RPM
0
5
10
M15
(295)
Relative averaged RPM
0
7.5
15
Anthesis
5DPA
10DPA
20DPA
30DPA
MG_stem
MG_equ
MG_stylar
Br_stem
Br_equ
Br_stylar
Pk_stem
Pk_equ
Pk_stylar
LR
RR
Total_pericarp
Septum
Locular_tissue
Placenta
Columella
Seeds
Outer_epidermis
Collenchyma
Parenchyma
Vascular_tissue
Inner_epidermis

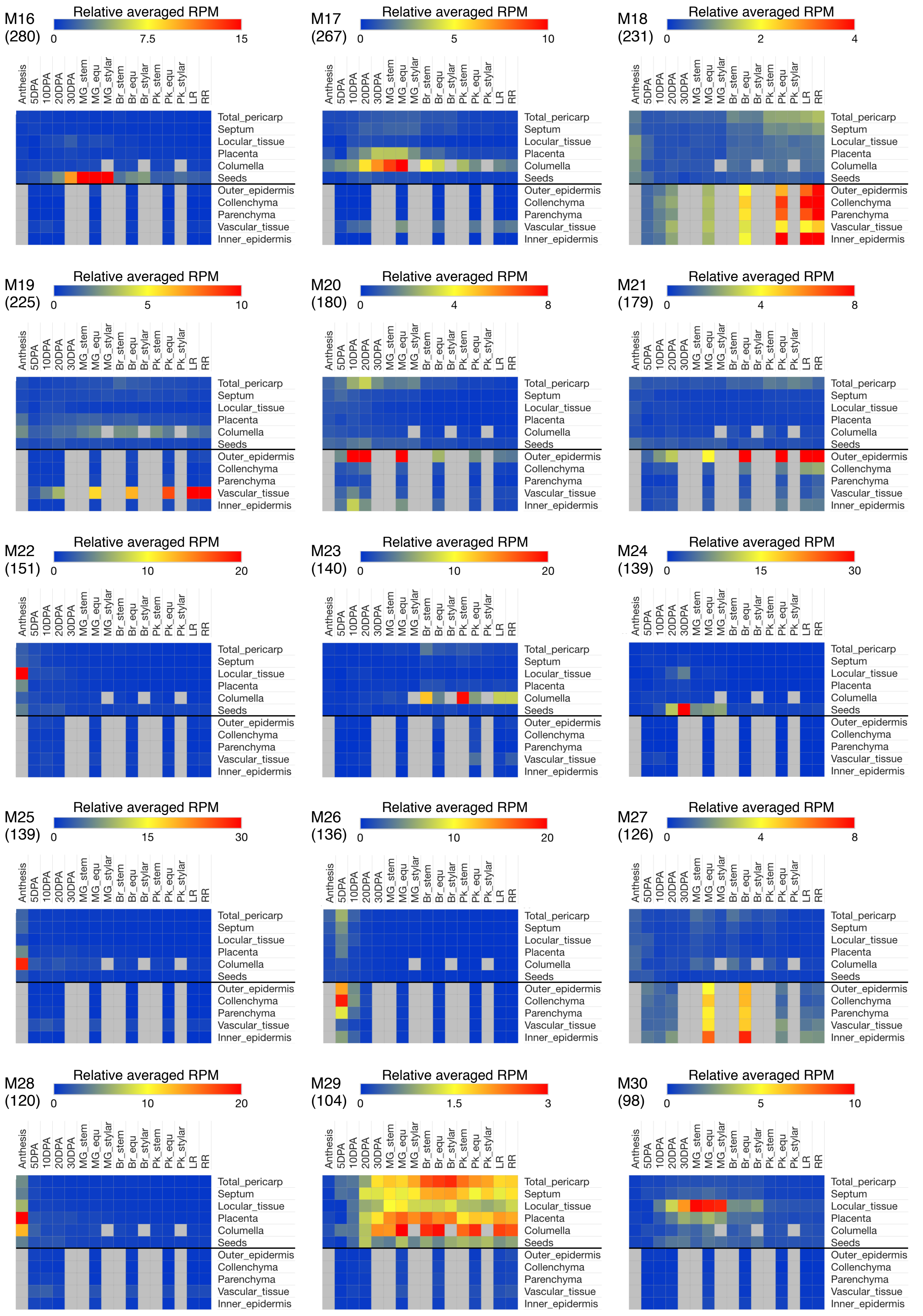
M16
(280)
Relative averaged RPM
0 7.5 15
M17
(267)
Relative averaged RPM
0 5 10
M18
(231)
Relative averaged RPM
0 2 4
M19
(225)
Relative averaged RPM
0 5 10
M20
(180)
Relative averaged RPM
0 4 8
M21
(179)
Relative averaged RPM
0 4 8
M22
(151)
Relative averaged RPM
0 10 20
M23
(140)
Relative averaged RPM
0 10 20
M24
(139)
Relative averaged RPM
0 15 30
M25
(139)
Relative averaged RPM
0 15 30
M26
(136)
Relative averaged RPM
0 10 20
M27
(126)
Relative averaged RPM
0 4 8
M28
(120)
Relative averaged RPM
0 10 20
M29
(104)
Relative averaged RPM
0 1.5 3
M30
(98)
Relative averaged RPM
0 5 10
Anthesis
5DPA
10DPA
20DPA
30DPA
MG_stem
MG_equ
MG_stylar
Br_stem
Br_equ
Br_stylar
Pk_stem
Pk_equ
Pk_stylar
LR
RR
Total_pericarp
Septum
Locular_tissue
Placenta
Columella
Seeds
Outer_epidermis
Collenchyma
Parenchyma
Vascular_tissue
Inner_epidermis

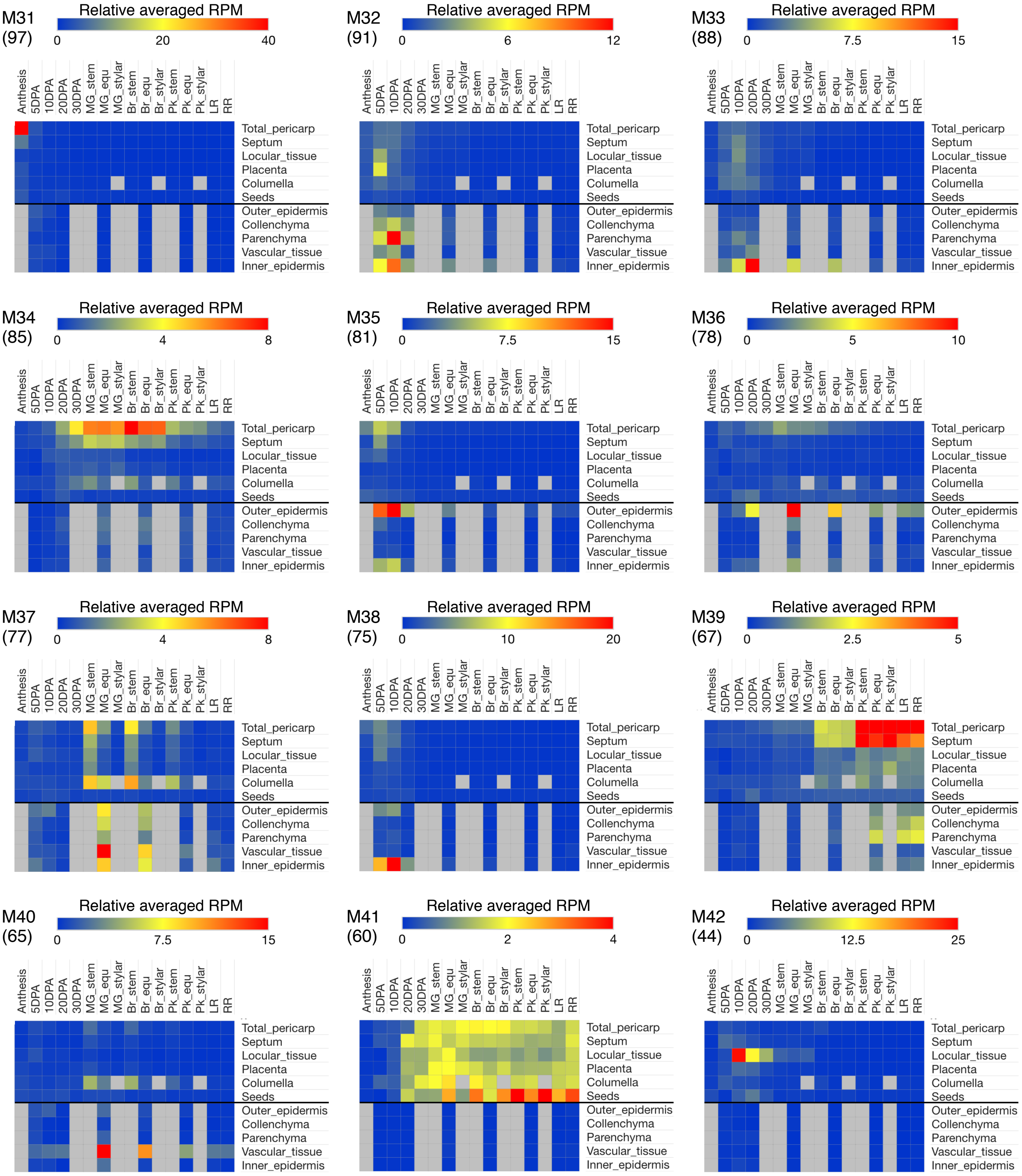

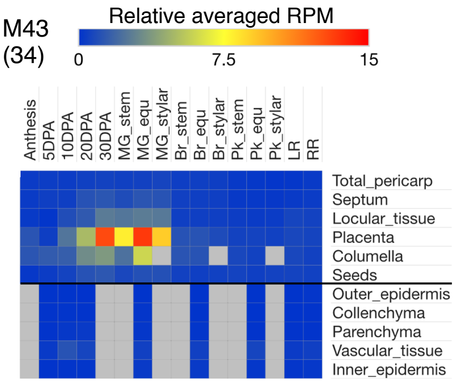

Supplementary Fig. 10 Heat maps showing averaged relative RPM values of all genes clustered in the WGCNA-generated co-expression modules M1-M43. The number of clustered genes is indicated by the values in parentheses. DPA, days post anthesis; MG, mature green; Br, breaker; Pk, pink; LR, light red; RR, red ripe; stem, stem-region; equ, equatorial-region; stylar, stylar-region.

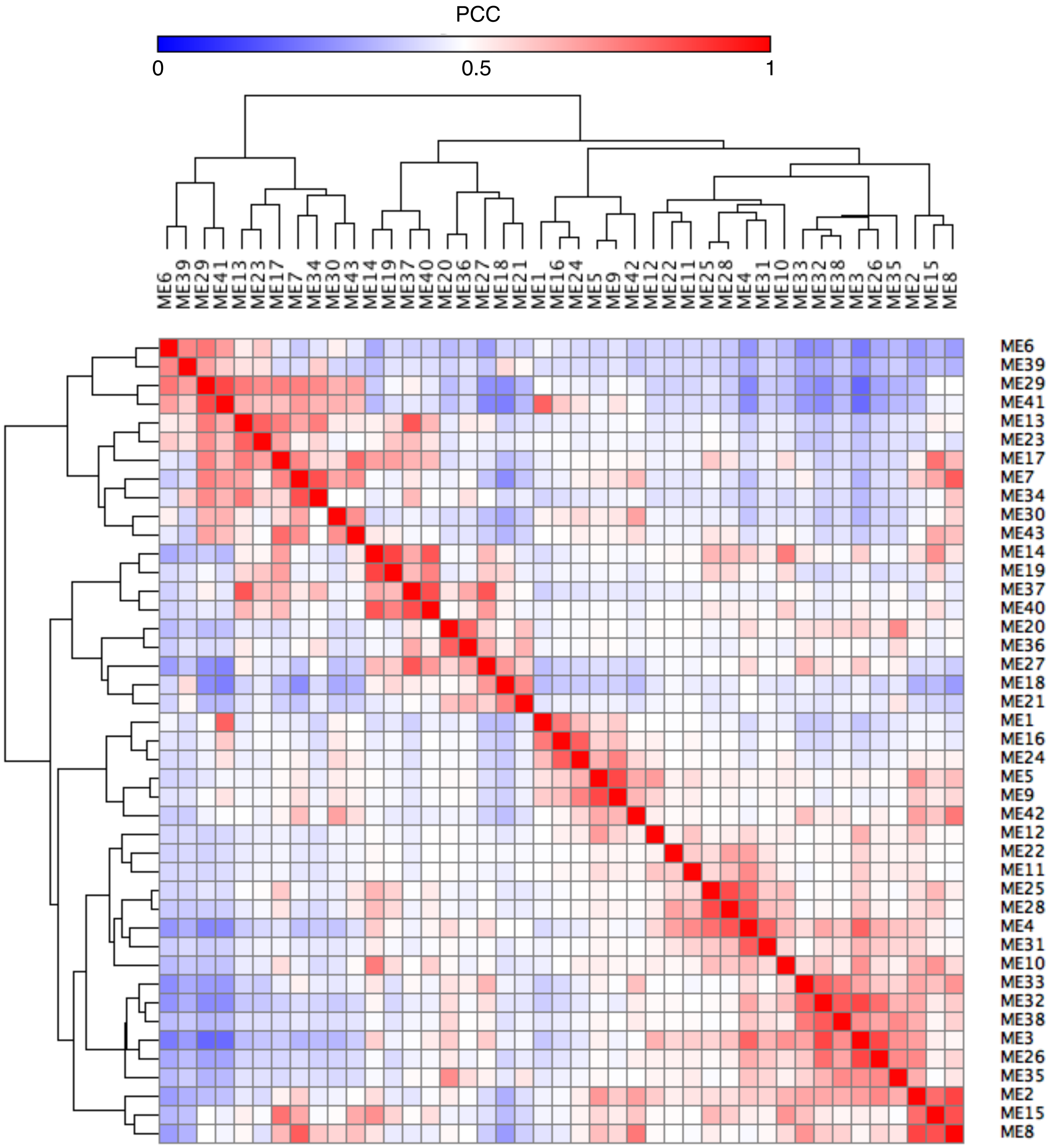

Supplementary Fig. 11 Module adjacencies based on correlations between module eigengene values. Each row/column in the heat map represent an module eigengene (ME). Red indicates strong positive correlation, blue indicates strong negative correlation and white indicates no correlation, as shown in the color scale bar. PCC, Pearson correlation coefficient.

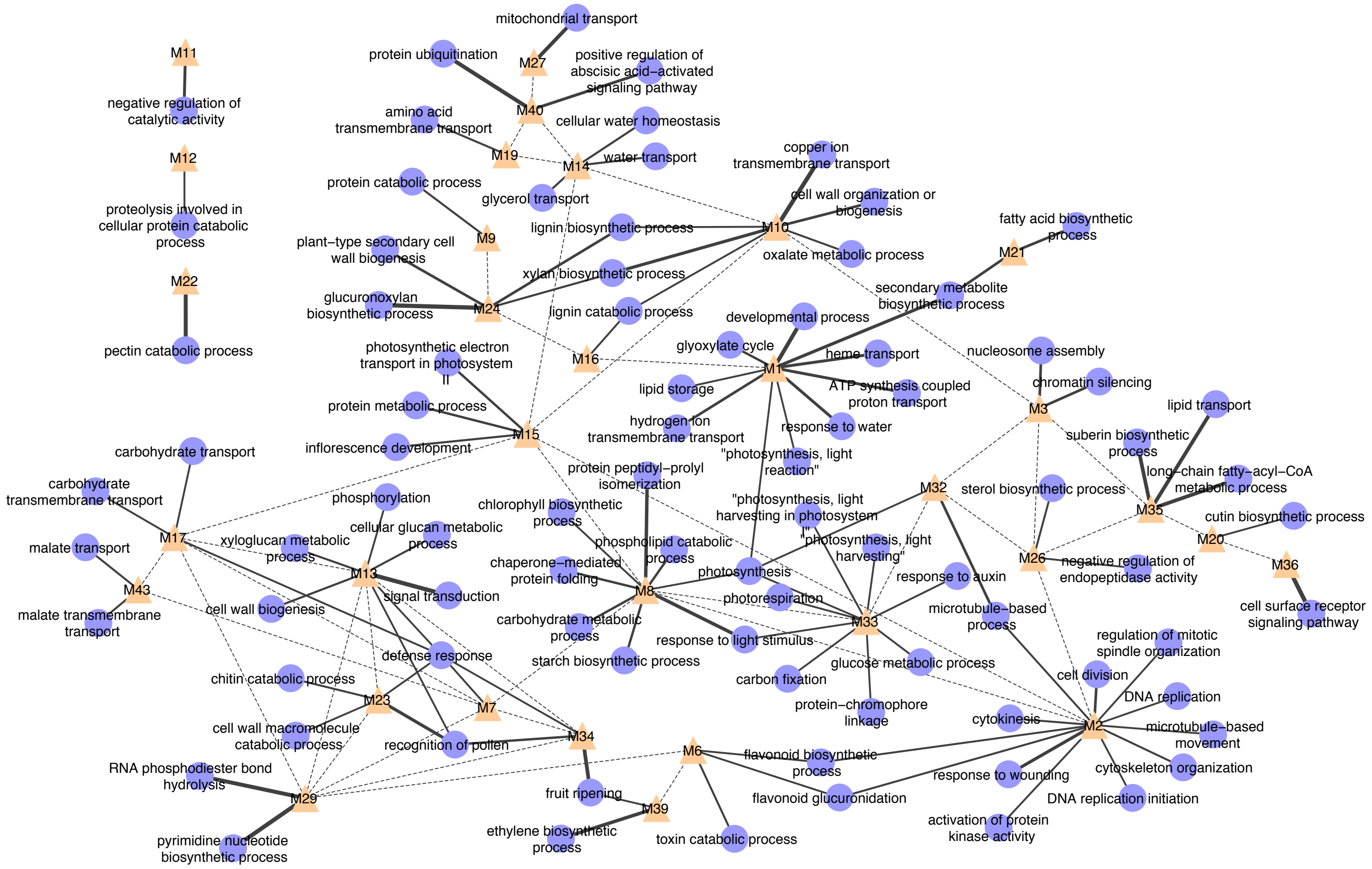

Supplementary Fig. 12 Module-gene ontology network of fruit cell/tissue development. The triangles and circles represent nodes of co-expression WGCNA modules and gene ontology (GO) terms (FDR-adjusted *p*-value < 0.01 , hypergeometric test), respectively. Edges with dashed lines represent intermodular correlation (PCC > 0.7). All pairwise intermodular correlations and enriched GO terms (FDR-adjusted *p*-value < 0.05, hypergeometric test) are shown in **Supplementary Fig. 11** and **Supplementary Data 8**, respectively.

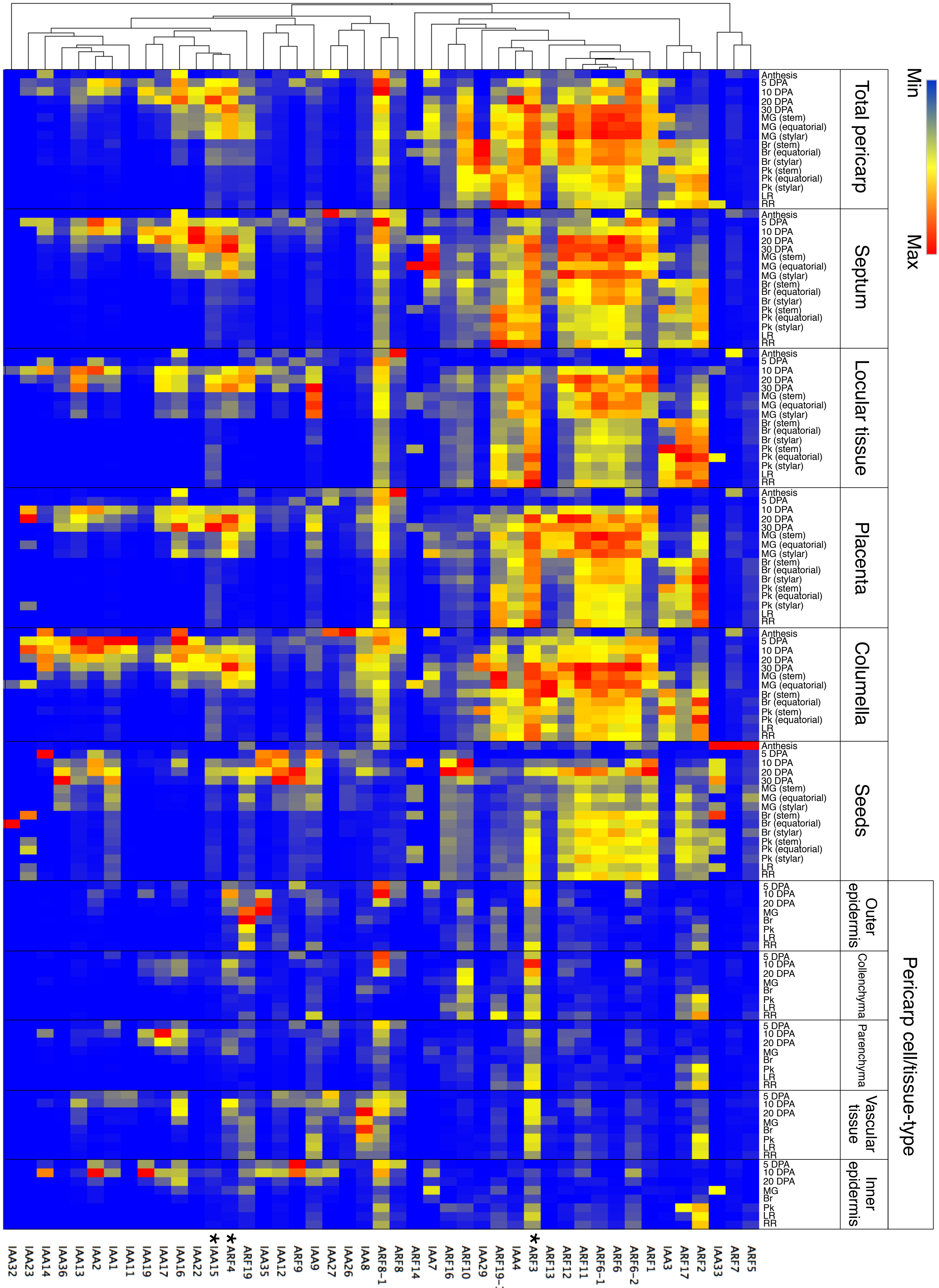

Supplementary Fig. 13 Heat map showing gene expression patterns of auxin signaling related *ARF* and *Aux/IAA* genes in tomato fruit tissues and cells during development. Genes analyzed by BiFC in this study are indicated by asterisks. DPA, days post anthesis; MG, mature green; Br, breaker; Pk, pink; LR, light red; RR, red ripe.

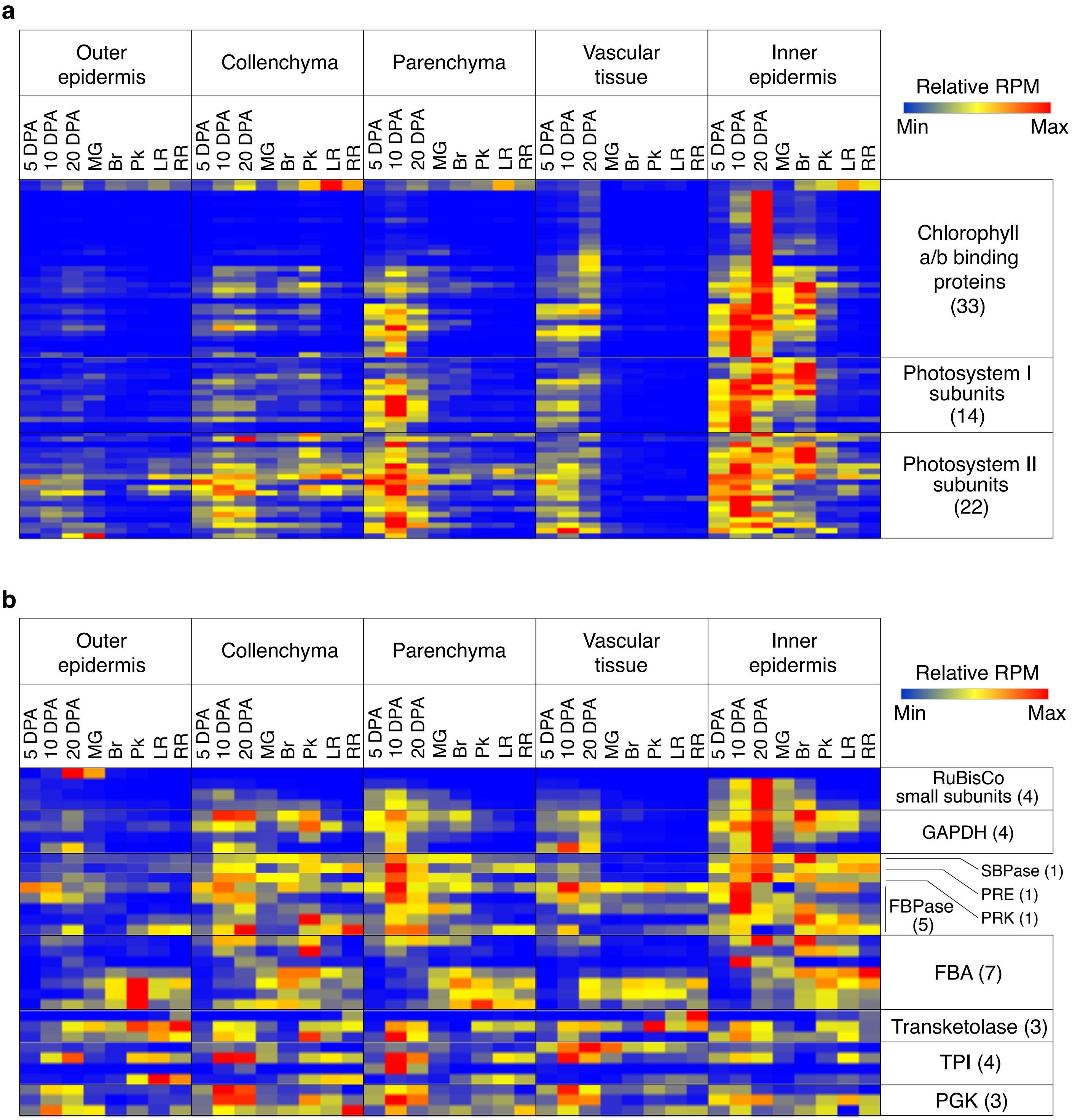

Supplemental Fig. 14 Heat map showing gene expression patterns of photosynthesis related genes in tomato pericarp cells during development. **a**, **b** The expression patterns of genes encoding proteins associated with light reaction **a** and Calvin cycle **b** are shown. The number of genes is indicated by the values in parentheses. RuBisCo, ribulose 1,5-bisphosphate carboxylase/oxygenase; GAPDH, glyceraldehyde-3-phosphate dehydrogenase; SBPase, sedoheptulose-bisphosphatase; PRE, pentose-5-phosphate 3-epimerase; PRK, phosphoribulokinase; FBPase, fructose 1,6-bisphosphatase; FBA, fructose-bisphosphate aldolase; TPI, triosephosphate isomerase; PGK, phosphoglycerate kinase. DPA, days post anthesis; MG, mature green; Br, breaker; Pk, pink; LR, light red; RR, red ripe.

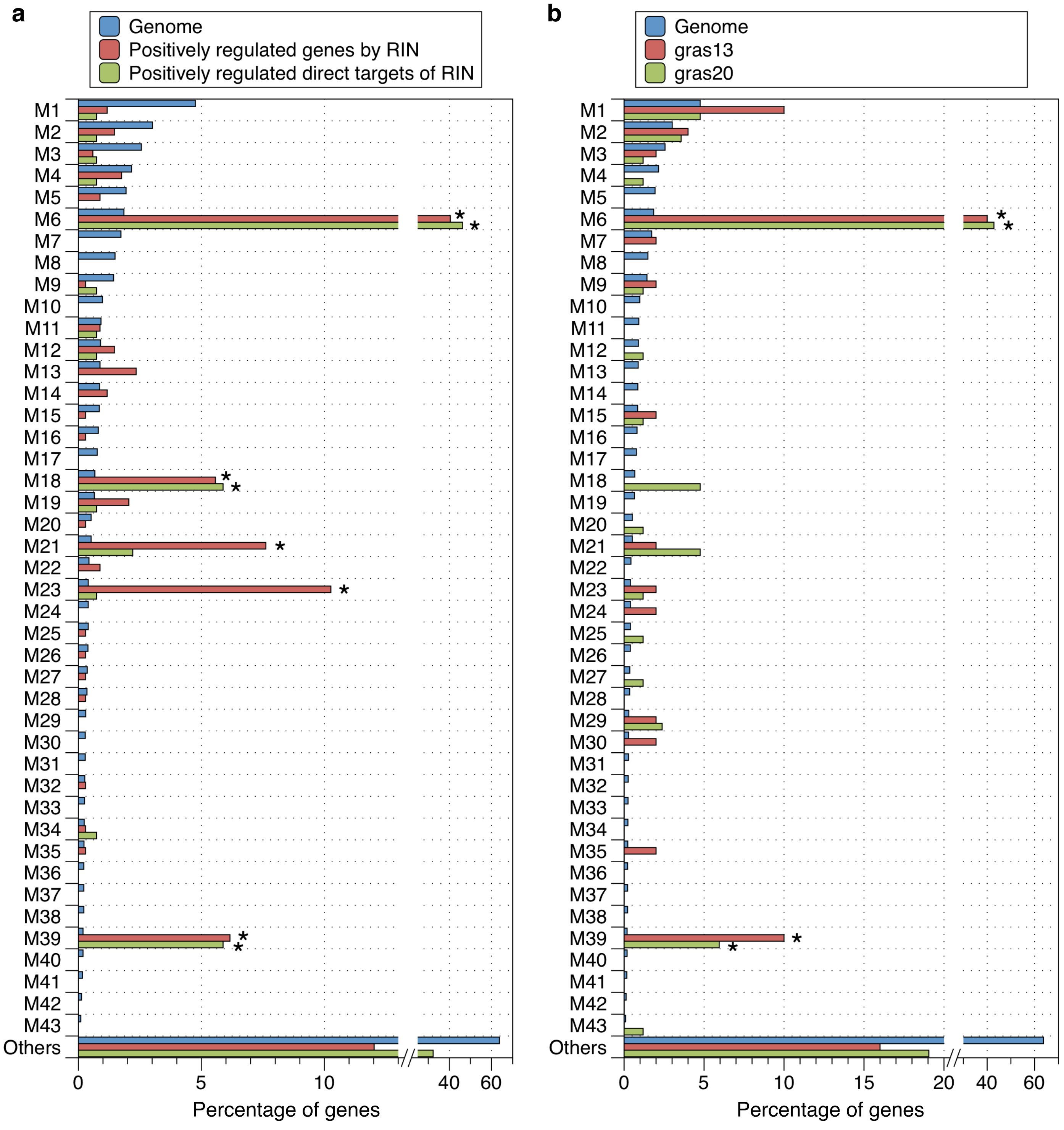

Supplementary Fig. 15 Module distribution of gene sets. **a** The percentages of 342 assigned genes that were reported as being positively regulated by RIN (Fujisawa et al., 2012)[1], or 137 positively regulated direct target genes of RIN (Fujisawa et al., 2013)[2] in each WGCNA module. **b** The percentages of assigned genes that were expressed at lower levels in *gras-13* (50 genes) or *gras-20* (84 genes) than in WT, in each WGCNA module. The proportions were compared to the respective frequencies in the genome. 'Others' includes genes not used for WGCNA and not clustered with any modules by the analysis. Asterisks indicate significant differences from the relative frequencies in the genome (FDR-adjusted p-value < 0.01, hypergeometric test).

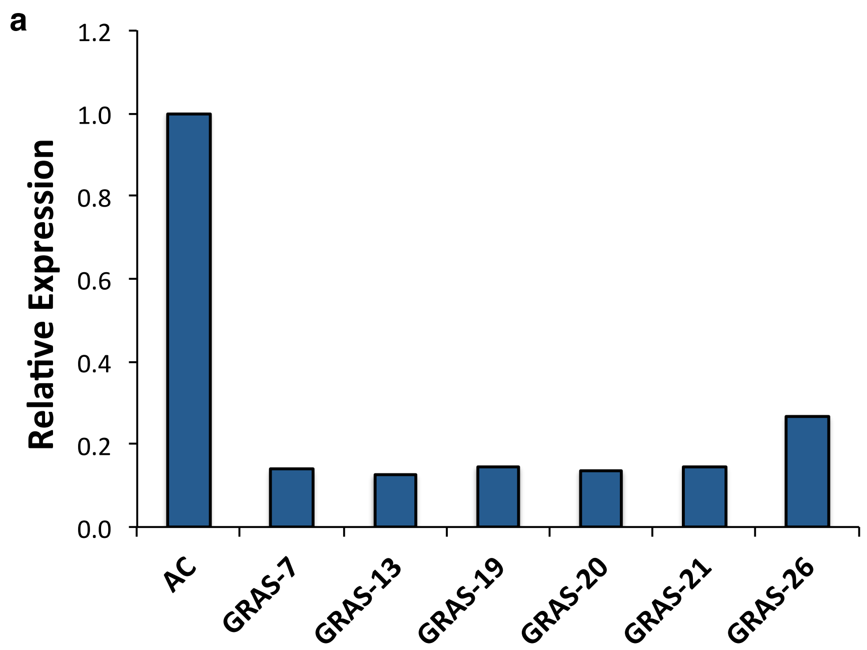

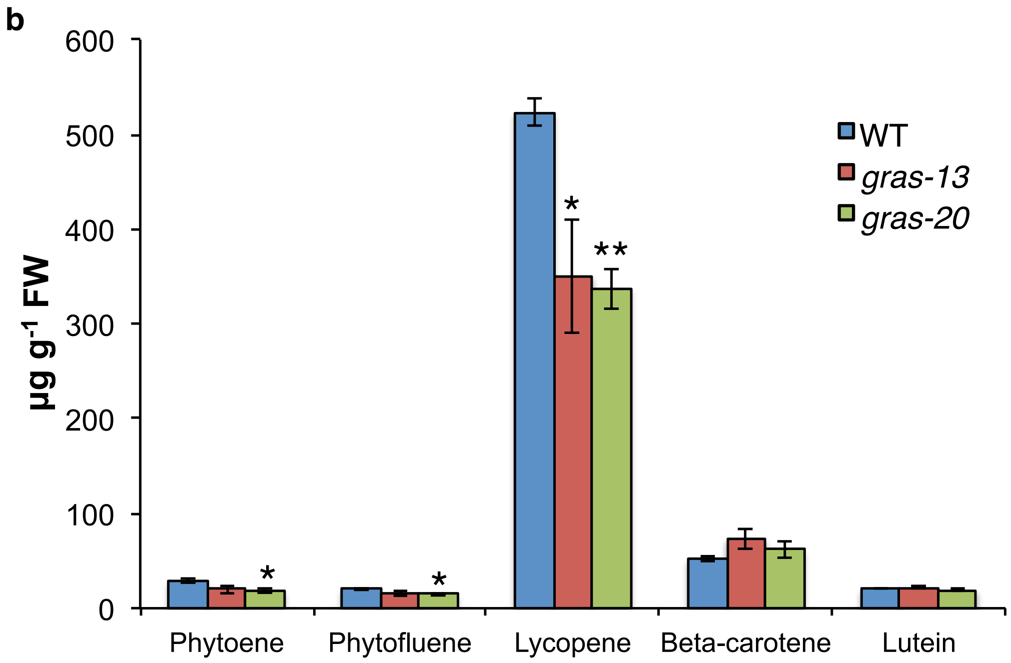

Supplementary Fig. 16 Additional gene expression and phenotypic data for *SlGRAS38*-RNAi lines. **a** Expression of *SlGRAS38* in Br-stage fruits from WT (cv Ailsa Craig) and five T0 *SlGRAS38*-RNAi lines (lines 7, 13, 19, 21 and 26); expression data for line 20 is from the T1 generation. Due to limited fruit set, analysis of T0 *SlGRAS38*-RNAi lines was conducted on single Br-stage fruit. All *SlGRAS38* expression values were normalized to *18S* and are shown relative to WT levels. **b** Pericarp carotenoid levels in *SlGRAS38*-RNAi lines and WT at 15 days after Br-stage. Values are mean ± s.e.m. (n = 3). Asterisks indicate significant differences between WT and each *SlGRAS38*-RNAi line (**$P < 0.01$, *$P < 0.05$; Student's *t*–test).

Please confirm.

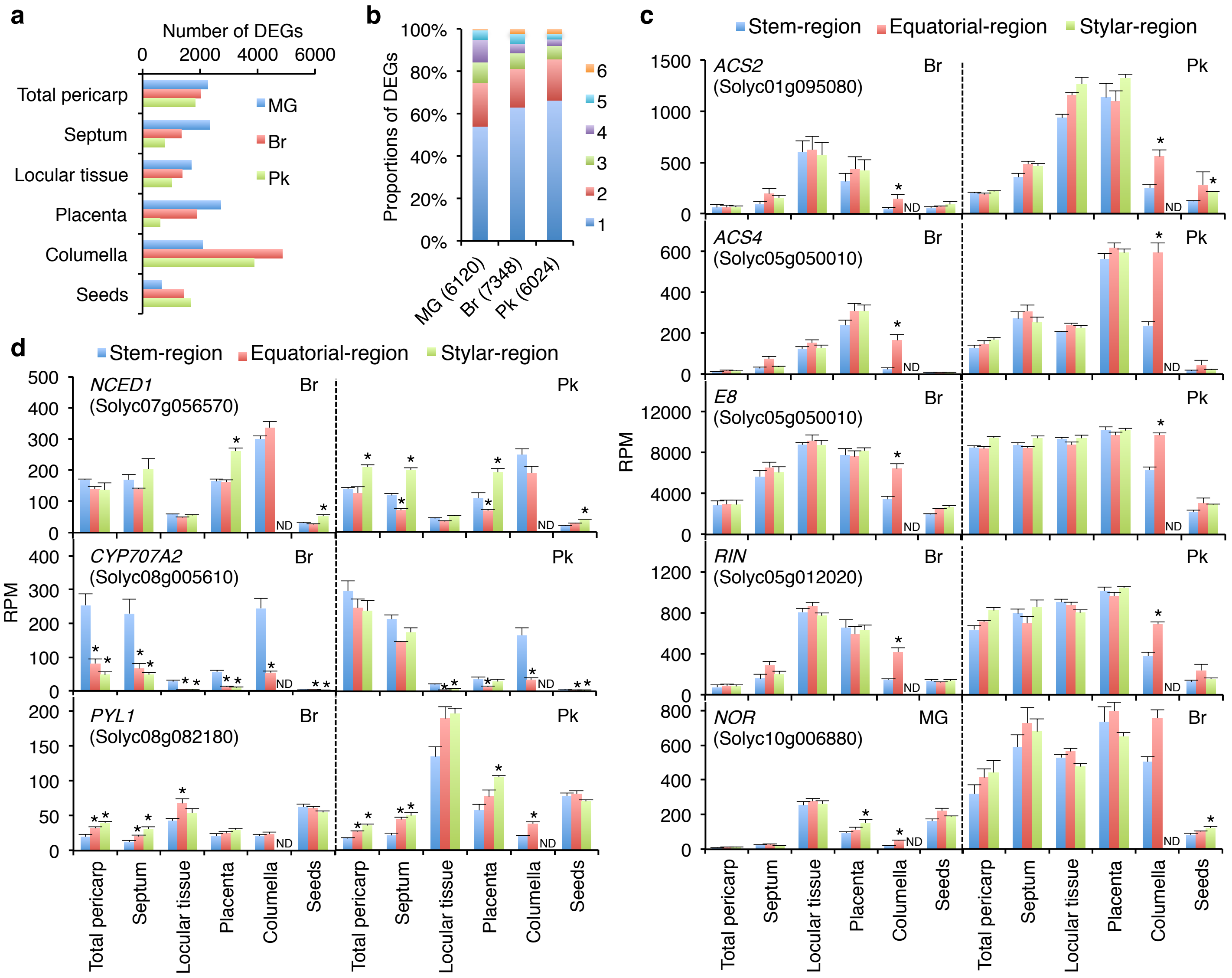

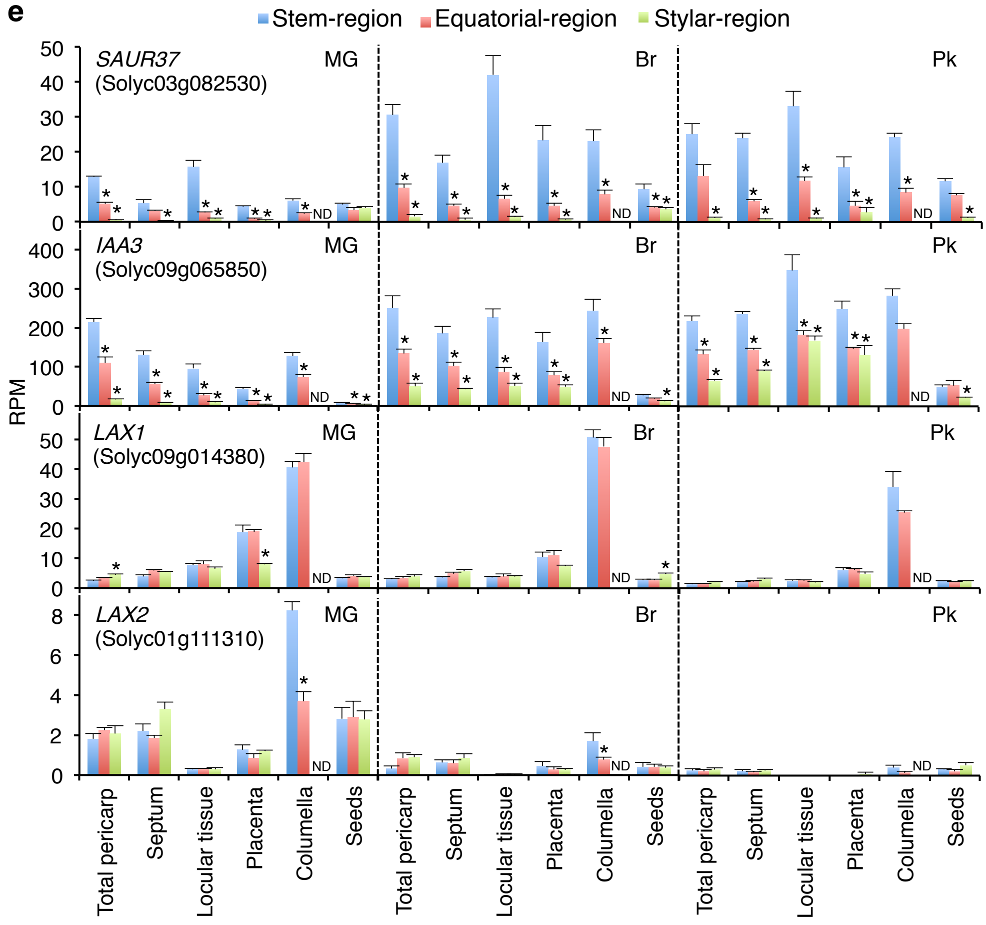

Supplementary Fig. 17 Differentially expressed genes along the latitudinal axis of tomato fruit during early ripening. **a** Number of differentially expressed genes (DEGs) between stem-region and stylar-region (total pericarp, septum, locular tissue, placenta, and seeds) or equatorial-region (columella) at MG, Br, and Pk stage (FDR-adjusted *p*-value < 0.05, exact test using edgeR package) with ratio ≥ 1.5 or ≤ 0.66. **b** Number of DEGs detected in one or more fruit tissues. Values in parentheses indicate total number of DEGs across all tissues at each stage. **c–e** Expressions of selected DEGs associated with ethylene (*ACS2*, *ACS4*, and *E8*) and ripening regulators (*RIN* and *NOR*) **c**, ABA biosynthesis (*NCED1*), catabolism (*CYP707A2*), and receptor (*PYL1*) **d**, and auxin response (*SAUR37* and *IAA3*) and influx transport (*LAX1* and *LAX2*) **e** during early ripening stages. Values are means ± s.e.m. of biological quadruplicates. Asterisks indicate significant differences from stem-region (FDR-adjusted *p*-value < 0.05, exact test using edgeR package) with ratio ≥ 1.5 or ≤ 0.66 . ND, no data.

Supplementary Table 1. Primer sequences used in this study.

Name	Sequence (5'-3')
For cloning fragments for *SlGRAS38* RNAi construct	
GRAS_attB1	GGGGACAAGTTTGTACAAAAAAGCAGGCTTAGCAGGAGCACTACAAGGA
GRAS_attB2	GGGGACCACTTTGTACAAGAAAGCTGGGTGTGAACTTTTGTTTGCCTTT
For qPCR	
GRAS_qPCR_F	GTTCCTTTTGAATTCCATGT
GRAS_qPCR_R	AAGTGCACCCTTAATTTGTT
RPL2_qPCR_F	CAGCGGATGTCGTGCTATGAT
RPL2_qPCR_R	GGGATGCTCCACTGGATTCA
18S_qPCR_F	CGGAGAGGGAGCCTGAGAA
18S_qPCR_R	CCCGTGTTAGGATTGGGTAATTT
For McrBC-PCR	
RIN_McrBC_Region1_F	TCAGCTTTCCAACGACAAAC
RIN_McrBC_Region1_R	GGAGTTTTCGATGAGCAACAC
RIN_McrBC_Region2_F	GTAGAATTTGGGGAAGAAACGTC
RIN_McrBC_Region2_R	TATCAATAGTCACATCCCCTTGTG
CNR_McrBC_F	TGAGCATCAACCACTCCTAATA
CNR_McrBC_R	CAGACTTAGTAATAACTCCGAT
PME_McrBC_F	AAACTAGACCATGAGTGTTGAGA
PME_McrBC_R	TTTTAGAGTGAATTACAGAGAAGC

Supplementary Table 2. Summary of RNA-seq mapping for *SlGRAS38*-RNAi lines.

Genotype	Replicates	Raw reads	Cleaned reads (removed adapter, low quality, and rRNA)	mapped	
				No. reads	%mapped
WT	1	5,930,115	5,103,428	4,307,965	84.4
	2	5,156,375	4,509,256	3,883,697	86.1
	3	5,071,402	4,407,592	3,562,552	80.8
gras13	1	5,054,194	3,510,511	2,963,123	84.4
	2	5,895,838	5,289,095	4,744,779	89.7
	3	5,772,147	5,246,030	4,566,363	87.0
gras20	1	4,893,350	4,167,174	3,265,753	78.4
	2	5,024,685	4,238,381	3,202,246	75.6
	3	5,473,332	4,397,368	3,517,184	80.0

Supplementary Table 3. Reproducibility of RNA-Seq reads for *SlGRAS38*-RNAi lines.

**Genotype**	**Spearman correlation coefficient values**					
	**Rep. 1 vs Rep. 2**	**Rep. 1 vs Rep. 3**	**Rep. 2 vs Rep. 3**	**Min**	**Max**	**Average**
WT	0.980	0.977	0.986	0.977	0.986	0.981
gras13	0.985	0.983	0.974	0.974	0.985	0.981
gras20	0.967	0.978	0.983	0.967	0.983	0.976

Supplementary Table 4. Summary of optimized conditions of the UltraPerformance Convergence Chromatography (UPC^2) system.

**Time (min)**	**Flow (mL/min)**	**% CO2**	**% MeOH**	**Curve**
Initial	1	99	1	Initial
7.5	1	80	20	9
12	1	80	20	6
15	1	99	1	1

Supplementary Note

Functional associations of WGCNA modules

Weighted gene co-expression network analysis (WGCNA)[3] using high resolution spatiotemporal dataset from developing tomato identified a total of 43 modules (M1 to M43), containing from 34 (M43) to 1,652 (M1) clustered genes (**Supplementary Fig. 10** and **Supplementary Data 7**). Intermodular similarities are shown (**Supplementary Fig. 11**) as Pearson correlations between module eigengene (ME) values that summarize the expression profiles of each module as the first principal component.

Gene ontology (GO) terms were assigned to genes in the various modules, which supported known aspects of tomato fruit biology. We also identified intramodular hub genes, which are defined by high ME-based gene connectivity (kME) scores (> 0.9), and have high regulatory and/or functional associations with constituent members of a particular module[4] (**Supplementary Data 8** and **9** and **Supplementary Fig. 12**). For example, among the modules related to particular fruit tissues, M39, which was associated with ripening of pericarp and septum, showed enrichment in the terms "fruit ripening" and "ethylene biosynthetic process", and included a hub gene encoding ACO1, key enzyme in the biosynthesis of the ripening-related hormone ethylene[5]. M1, associated with late developing seeds, showed enrichment in "lipid storage[6]" and included multiple hub genes encoding oleosin[7] and late embryogenesis abundant proteins (LEA)[8], as well as an abscisic acid (ABA) biosynthetic enzyme, FLACCA, and an ABA signaling transcription factor, ABI3, which are associated with seed maturation and dormancy[9,10]. Modules showing preferential expression in the columella, had a high frequency of the terms "lignin biosynthetic process" (M15), "carbohydrate transport" and "sulfate transport" (M17), consistent with the high abundance of vascular bundles in this tissue (**Supplementary Fig. 7c** and **d**).

Modules representing expression in a particular pericarp cell/tissue-type included enriched GO terms and hub genes reflecting cell specialization (**Supplementary Data 8** and **9**). For example, modules with preferential expression in the outer epidermis between 5 DPA and 10 DPA (M35), or 10 DPA and MG (M20), have many genes annotated with "long-chain fatty-acyl-CoA metabolic process" and "cutin biosynthetic process", respectively. This reflects the biosynthesis of the cutin-rich hydrophobic outer epidermal cuticle during fruit expansion[11,12]. Hub genes of M35 included a homolog (Solyc05g009270) of *LeCER6*[13], which encodes a very-long-chain fatty acid β-ketoacyl-CoA synthase . M20 also included the cutin biosynthetic genes *CUTIN DEFICIENT 1* (*CD1*)/*SlCUS1*[14], *CD3*/*SlCYP86A69*[15] and *SlGPAT6*[16] as hubs. M10, which contains genes expressed in the pericarp vascular tissue during early fruit growth, is enriched in "lignin biosynthetic process" genes, and includes homologs (Solyc09g011960, Solyc02g085110 and Solyc02g062650) of *A. thaliana* laccase genes that are involved in monolignol polymerization during lignin formation[17]. We also found that the "amino acid transmembrane transport" category is linked with pericarp vascular tissue (M19) specifically during fruit ripening. Hubs in this module include two genes (Solyc06g060110 and Solyc01g106800) that encode homologs of *A. thaliana* AMINO ACID PERMEASE 3 (AtAAP3) and AtAAP6, respectively, which both transport a broad range of amino acids[18,19]. Metabolism of amino acids in ripening tomato fruit makes important contributions to fruit taste, flavor and nutritional quality[20,21]. Cell/tissue-type, or developmental stage specific, co-expression of genes associated with amino acid transport likely contribute to the substantial changes in the content of free amino acids within the ripening pericarp. These can accumulate in the fruit by translocation from the leaves, or generated by *in situ* metabolism[22-25].

Our results revealing spatiotemporally co-expressed modules provide a valuable platform for the identification and functional verification of the regulatory gene networks controlling fruit cell/tissue biology.

Supplementary References

1. Fujisawa, M. *et al.* Direct targets of the tomato-ripening regulator RIN identified by transcriptome and chromatin immunoprecipitation analyses. *Planta* **235,** 1107–1122 (2012).
2. Fujisawa, M., Nakano, T., Shima, Y. & Ito, Y. A large-scale identification of direct targets of the tomato MADS box transcription factor RIPENING INHIBITOR reveals the regulation of fruit ripening. *Plant Cell* **25,** 371–386 (2013).
3. Langfelder, P. & Horvath, S. WGCNA: an R package for weighted correlation network analysis. *BMC Bioinformatics* **9,** 559 (2008).
4. Langfelder, P., Luo, R., Oldham, M. C. & Horvath, S. Is my network module preserved and reproducible? *PLoS Comput. Biol.* **7,** e1001057 (2011).
5. Van de Poel, B. *et al.* Tissue specific analysis reveals a differential organization and regulation of both ethylene biosynthesis and E8 during climacteric ripening of tomato. *BMC Plant Biol.* **14,** 11 (2014).
6. Belmonte, M. F. *et al.* Comprehensive developmental profiles of gene activity in regions and subregions of the *Arabidopsis* seed. *Proc. Natl. Acad. Sci. U.S.A.* **110,** E435–E444 (2013).
7. Siloto, R. M. P. The accumulation of oleosins determines the size of seed oilbodies in *Arabidopsis*. *Plant Cell* **18,** 1961–1974 (2006).
8. Olvera-Carrillo, Y., Reyes, J. L. & Covarrubias, A. A. Late embryogenesis abundant proteins. *Plant Signal. Behav.* **6,** 586–589 (2011).
9. Weyers, J. D. B. Germination and root gravitropism of *flacca*, the tomato mutant deficient in abscisic acid. *J. Plant Physiol.* (1985). doi:10.1016/S0176-1617(85)80083-3
10. Gao, Y. *et al.* Functional characterization of two alternatively spliced transcripts of tomato *ABSCISIC ACID INSENSITIVE3* (*ABI3*) gene. *Plant Mol. Biol. Rep.* **82,** 131–145 (2013).
11. Segado, P., Domínguez, E. & Heredia, A. Ultrastructure of the epidermal cell wall and cuticle of tomato fruit (*Solanum lycopersicum* L.) during development. *Plant Physiol.* **170,** 935–946 (2016).
12. Martin, L. B. B. & Rose, J. K. C. There's more than one way to skin a fruit: formation and functions of fruit cuticles. *J. Exp. Bot.* **65,** 4639–4651 (2014).
13. Vogg, G. *et al.* Tomato fruit cuticular waxes and their effects on transpiration barrier properties: functional characterization of a mutant deficient in a very-long-chain fatty acid beta-ketoacyl-CoA synthase. *J. Exp. Bot.* **55,** 1401–1410 (2004).
14. Yeats, T. H. *et al.* The identification of cutin synthase: formation of the plant polyester cutin. *Nat. Chem. Biol.* **8,** 609–611 (2012).
15. Shi, J. X. *et al.* The tomato SlSHINE3 transcription factor regulates fruit cuticle formation and epidermal patterning. *New Phytol.* **197,** 468–480 (2013).
16. Petit, J. *et al.* The Glycerol-3-Phosphate Acyltransferase GPAT6 from Tomato Plays a Central Role in Fruit Cutin Biosynthesis. *Plant Physiol.* **171,** 894–913 (2016).
17. Zhao, Q. *et al. LACCASE* is necessary and nonredundant with *PEROXIDASE* for lignin polymerization during vascular development in *Arabidopsis*. *Plant Cell* **25,** 3976–3987 (2013).
18. Breitkreuz, K. E., Shelp, B. J., Fischer, W. N., Schwacke, R. & Rentsch, D. Identification and characterization of GABA, proline and quaternary ammonium compound transporters from *Arabidopsis thaliana*. *FEBS Lett.* **450,** 280–284 (1999).
19. Fischer, W. N. *et al.* Low and high affinity amino acid H+-cotransporters for cellular import of neutral and charged amino acids. *Plant J.* **29,** 717–731 (2002).

20. Bellisle, F. Glutamate and the UMAMI taste: sensory, metabolic, nutritional and behavioural considerations. A review of the literature published in the last 10 years. *Neurosci. Biobehav. Rev.* **23,** 423–438 (1999).
21. Klee, H. J. & Giovannoni, J. J. Genetics and control of tomato fruit ripening and quality attributes. *Annu. Rev. Genet.* **45,** 41–59 (2011).
22. Valle, E. M., Boggio, S. B. & Heldt, H. W. Free amino acid composition of phloem sap and growing fruit of *Lycopersicon esculentum*. *Plant Cell Physiol.* **39,** 458–461 (1998).
23. Boggio, S. B., Palatnik, J. F., Heldt, H. W. & Valle, E. M. Changes in amino acid composition and nitrogen metabolizing enzymes in ripening fruits of *Lycopersicon esculentum* Mill. *Plant Sci.* **159,** 125–133 (2000).
24. Do, P. T., Prudent, M., Sulpice, R., Causse, M. & Fernie, A. R. The influence of fruit load on the tomato pericarp metabolome in a *Solanum chmielewskii* introgression line population. *Plant Physiol.* **154,** 1128–1142 (2010).
25. Oms-Oliu, G. *et al.* Metabolic characterization of tomato fruit during preharvest development, ripening, and postharvest shelf-life. *Postharvest Biol. Tec.* **62,** 7–16 (2011).
