# Supplementary material for: High-resolution spatiotemporal transcriptome mapping of tomato fruit development and ripening
Source: Nat Commun. 2018 Jan 25;9:364. doi: 10.1038/s41467-017-02782-9 (PMC5785480; doi:10.1038/s41467-017-02782-9)
Supplement: Supplementary file 2 — Description of Additional Supplementary Files [file 41467_2017_2782_MOESM2_ESM.pdf]

Descriptions of Additional Supplementary Files

File Name: Supplementary Data 1
Description: Summary of RNA-Seq mapping for hand-dissected samples.

File Name: Supplementary Data 2
Description: Summary of RNA-Seq mapping for LM samples.

File Name: Supplementary Data 3
Description: Gene expression in fruit tissue/cell-types.

File Name: Supplementary Data 4
Description: Standard errors of gene expression in fruit tissue/cell-types.

File Name: Supplementary Data 5
Description: Reproducibility of RNA-Seq reads for hand-dissected samples.

File Name: Supplementary Data 6
Description: Reproducibility of RNA-Seq reads for LM samples.

File Name: Supplementary Data 7
Description: Module assignment and ME-based gene connectivity for the 12,662 genes selected for WGCNA.

File Name: Supplementary Data 8
Description: Gene ontology enrichment in co-expression WGCNA modules.

File Name: Supplementary Data 9
Description: Hub genes in co-expression WGCNA modules.

File Name: Supplementary Data 10
Description: Down-regulated genes in *SlGRAS38*-RNAi lines.

File Name: Supplementary Data 11
Description: Differentially expressed genes in the tissues among latitudinal fruit sections.
